# Real-time assessment of inter and intraventricular synchrony in cardiac resynchronization therapy with left bundle branch pacing using ECGi and low-frequency QRS analysis

**DOI:** 10.1007/s10840-026-02288-9

**Published:** 2026-04-14

**Authors:** Clara Sales-Belles, Ana Mincholé, Mercedes Cabrera-Ramos, Jorge Melero-Polo, Esther Pueyo, Javier Ramos Maqueda

**Affiliations:** 1 BSICoS Group, Aragon Institute of Engineering Research, IIS Aragón, University of Zaragoza, Zaragoza, Spain; 2 Arrhythmias Unit, Cardiology Department, Lozano Blesa Clinical University Hospital, Zaragoza, Spain; 3 CIBER de Bioingeniería, Biomateriales y Nanomedicina, Instituto de Salud Carlos III, Madrid, Spain; 4 Aragon Health Research Institute, Zaragoza, Spain; 5 Department of Medicine, Psychiatry and Dermatology, Faculty of Medicine, Universidad de Zaragoza, Zaragoza, Spain

**Keywords:** Left bundle branch pacing, ECG, electrocardiographic imaging, cardiac resynchronization therapy

## Abstract

**Introduction:**

Left bundle branch pacing (LBBP) is an alternative to conventional biventricular pacing for cardiac resynchronization therapy (CRT).

**Methods:**

A 76-year-old male was referred for CRT via left bundle branch pacing (LBBP). We present the real-time characterization of the transition from intrinsic left bundle branch block (LBBB) to sequential pacing modalities: right ventricular septal pacing (RVSP), left ventricular septal pacing (LVSP), and finally LBBP. Epicardial activation maps from electrocardiographic imaging (ECGi) and low-frequency (LF) QRS analysis were used to provide complementary insights into inter- and intraventricular synchrony.

**Results:**

LVSP, LBBP, and anodal capture improved activation compared with baseline, with LBBP achieving the greatest improvements in interventricular and LV intraventricular synchrony despite higher RV dispersion.

**Conclusion:**

These findings highlight ECGi and LF QRS as valuable tools to guide CRT.

**Graphical Abstract:**

Body surface potentials were recorded using a 128-electrode array covering the anterior and posterior torso. Ventricular ECGi maps and derived markers were then computed. A standard 12-lead ECG was simultaneously obtained from 10 conventional electrodes, and the ventricular activation sequence and precordial activation delay (pAD) were calculated. Both baseline and different pacing locations were analyzed.

**Figure:**
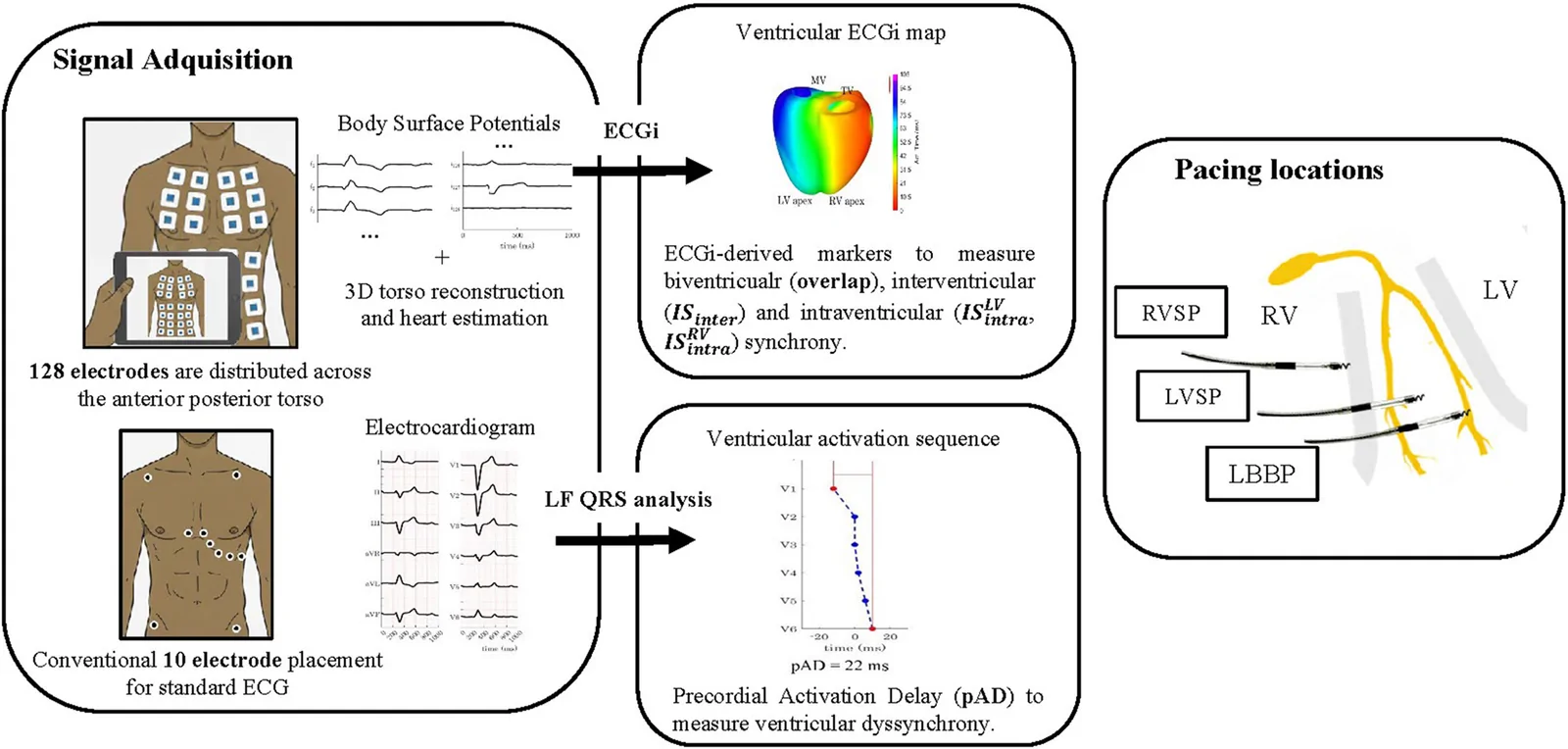

**Supplementary Information:**

The online version contains supplementary material available at https://doi.org/10.1007/s10840-026-02288-9.

## Introduction

Left bundle branch area pacing (LBBAP) has emerged as an alternative to conventional biventricular pacing for cardiac resynchronization therapy (CRT). This technique encompasses two main approaches: left bundle branch pacing (LBBP) and left ventricular septal pacing (LVSP). The key distinction between them lies in lead placement: LBBP involves direct capture of the left bundle branch, while LVSP stimulates the left ventricular septal myocardium without engaging the conduction system [1].

Electrocardiographic imaging (ECGi) is a noninvasive cardiac mapping technique that enables evaluation of epicardial ventricular activation [2] and has been used to guide lead positioning in pacing therapies [3]. Low-frequency (LF) QRS analysis, a method based on surface electrocardiogram (ECG) signals, has been used to derive activation sequences and estimate activation times from precordial leads [4].

In this report, we present a real-time characterization of the transition from baseline left bundle branch block (LBBB) to right ventricular septal pacing (RVSP), followed by LVSP, and ultimately LBBP, as the lumenless lead progressively penetrates the septum. Using ECGi for spatial mapping and LF QRS analysis for global activation assessment, we quantitatively evaluate the interventricular and intraventricular synchrony associated with each pacing modality.

## Case report

A 76-year-old male patient with permanent atrial fibrillation and rapid ventricular response refractory to optimal doses of beta-blockers and digoxin therapy was referred for CRT via LBBP and atrioventricular node ablation. He presented LBBB with a QRS duration of 153 ms, a left ventricular ejection fraction (LVEF) of 40%, and New York Heart Association (NYHA) functional Class III symptoms.

Prior to device implantation, the patient underwent ECGi using a 128-electrode vest (ACORYS system, Corify Care, Madrid). Torso geometry and electrode positions were acquired non-invasively via video recording and reconstructed using 3D photogrammetry [5]. Cardiac geometry was estimated based on patient weight and height. A standard 12-lead ECG was also recorded at a sampling frequency of 1000 Hz.

During implantation, a 3830 lumenless lead (Medtronic, Inc., Minneapolis, MN) was advanced into the right ventricular (RV) septum while monitoring for the characteristic “W pattern” in lead V1. The lead was screwed into the RV septum until reaching the left ventricular (LV) septum, at which point the “W” notch in lead V1 transitioned to a right bundle branch block (RBBB) pattern. LBBP was confirmed according to established criteria [1], defined by a V6RWPT < 80 ms [6] and a V6–V1 interpeak interval > 44 ms [7]. LVSP was identified by the presence of a qr pattern in lead V1 that did not meet these thresholds. Sequential pacing configurations were evaluated: RVSP, LVSP, LBBP, and LBBP with anodal capture.

**Fig. 1:**
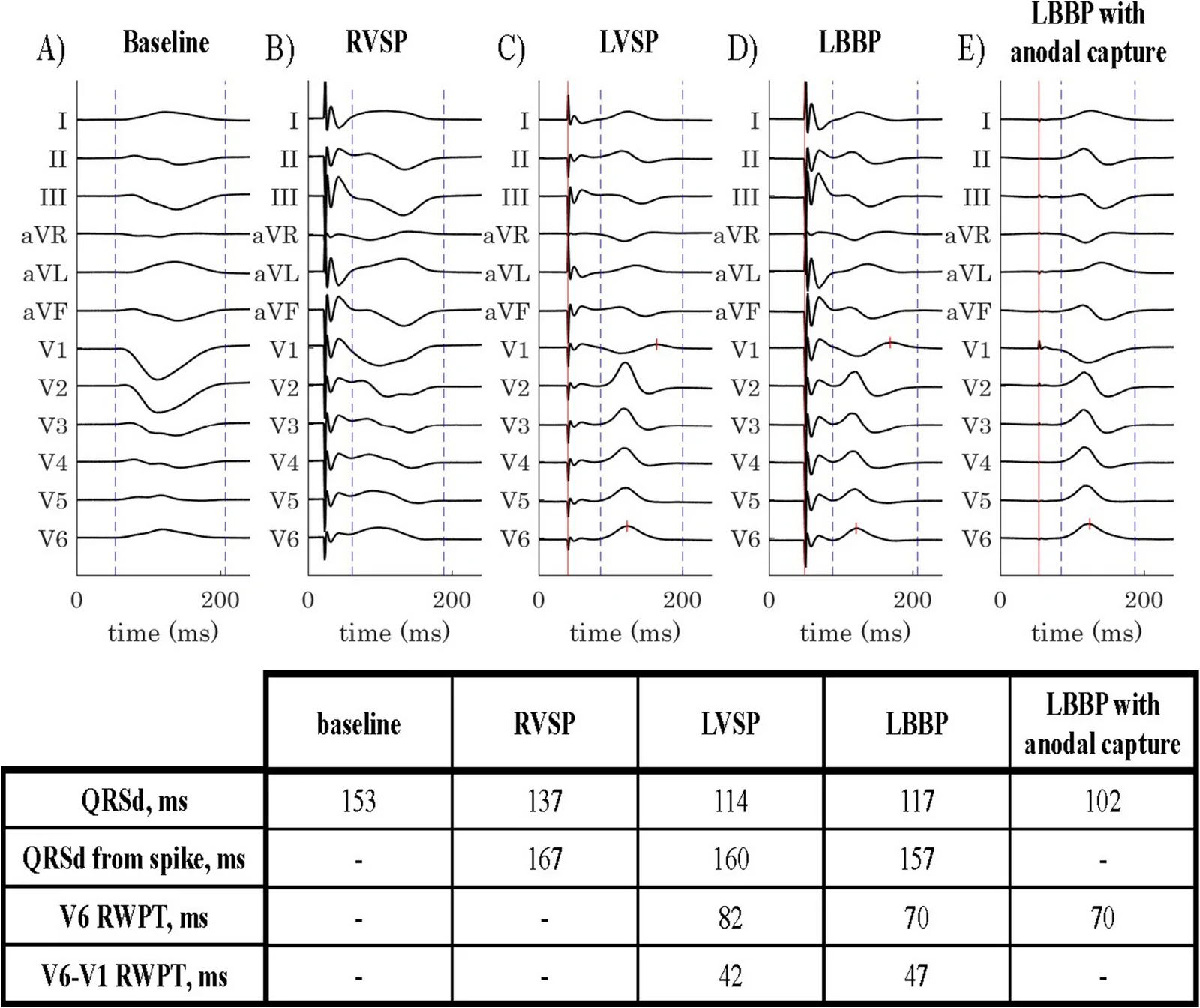
Standard 12-lead ECG for (**A**) baseline, (**B**) RVSP, (**C)** LVSP, (**D**) LBBP and (**E)** LBBP with anodal capture (AC) and QRSd, V6 RWPT and V1-V6 RWPT values. Dashed blue lines indicate the QRS complex boundaries. The red vertical line across all leads marks the pacing spike onset. Red line in V1and V6 indicate V1 and V6 R-wave peak

Standard 12-lead ECG markers were assessed, including QRS duration (QRSd), V6 R-wave peak time (V6RWPT), and the V1-V6 peak interval. V6RWPT was measured from the onset of the pacing spike to the peak of the R-wave in lead V6. The V1-V6 peak interval was calculated as the difference between V6RWPT and the interval from the pacing spike onset to the R’-wave peak in lead V1. These markers were measured at baseline and for each pacing modality (see Fig. 1).

ECGi-derived epicardial activation maps were analyzed to compute total activation time (AT_t_) as well as the total activation times for the right (AT_RV_) and left (AT_LV_) ventricles individually. These were defined as the 90th percentile of activation times over their respective (biventricular, right ventricular, and left ventricular) surfaces. Interventricular synchrony (${IS}_{inter}^{}$) was calculated as the difference in median activation times between the LV and RV, while intraventricular synchrony was defined for LV (${IS}_{intra}^{LV}$) and RV (${IS}_{intra}^{RV}$) as the difference between the 90th and 10th percentiles of activation times within each ventricle. Histograms of activation times for each ventricle were used to compute a biventricular synchrony index based on histogram overlap, with probability density functions fitted for visualization.

LF QRS analysis [4] was used to derive activation sequences and precordial activation delay (pAD) from precordial ECG leads V1-V6. In brief, this technique applies a low-pass filtering (10–60 Hz) to the QRS complex. Activation times are defined as the time point at which 50% of the cumulative area under the filtered QRS signal is reached for each lead. pAD is subsequently computed as the difference between the first and last activated leads and provides information on ventricular activation synchrony and direction. Low absolute values of pAD reflect fast and synchronized activation, while its sign is indicative of the directionality: positive pAD indicates delayed LV activation, whereas negative pAD indicates delayed RV activation.

Results are presented in the following. Standard ECG findings showed that RVSP was identified by a ‘W pattern’ in V1 and QRSd of 137 ms. LVSP criteria was met with V6RWPT of 82 ms, V1-V6 peak interval of 42 ms, and QRSd of 114 ms. LBBP was obtained with V6RWPT of 70 ms, V1-V6 peak interval of 47 ms, and QRSd of 117 ms. Once left bundle branch capture was achieved, bipolar pacing was performed, resulting in anodal capture, characterized by the absence of qR pattern in V1, QRSd of 102 ms, and V6RWPT of 70 ms (Fig. 1).

**Fig. 2:**
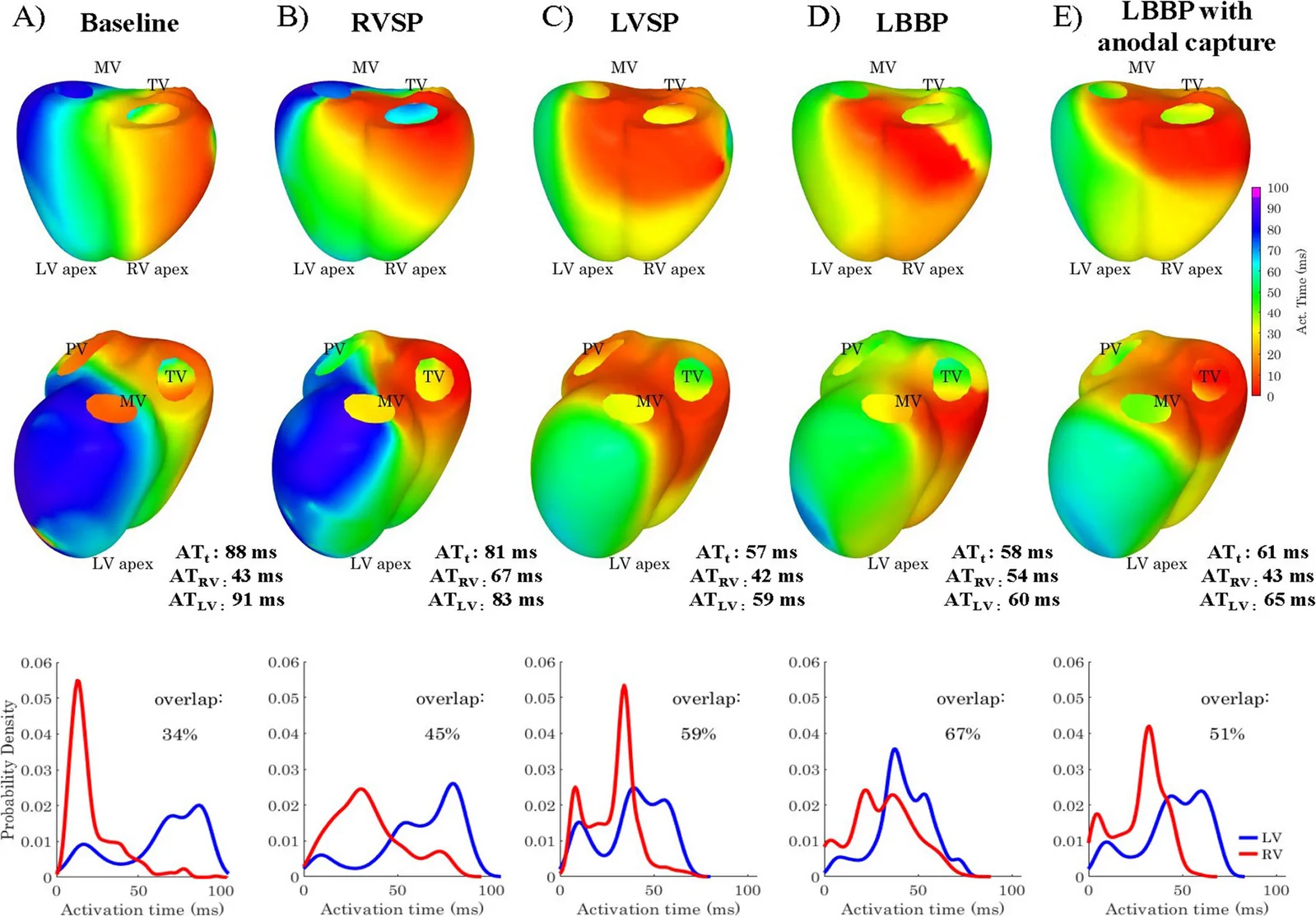
Epicardial activation maps with AT_t_, AT_RV_ and AT_LV_ values and probability density estimation of LV (blue) and RV (red) activation times for (**A**) baseline, (**B**) RVSP, (**C**) LVSP, (**D**) LBBP and (**E**) LBBP with anodal capture

ECGi analysis revealed delayed LV lateral wall activation at baseline and during RVSP, with AT_t_ values of 88 ms and 81 ms, respectively. In contrast, LVSP, LBBP, and anodal capture effectively reversed the LBBB pattern, showing early septal activation with rapid LV activation and AT_t_ values of 57 ms, 58 ms and 61 ms, respectively (Fig. 2). ${IS}_{inter}^{}$, ${IS}_{intra}^{RV}$ and ${IS}_{intra}^{LV}$markers under different pacing conditions are shown in Table 1. ${IS}_{inter}^{}$was markedly reduced from 54 ms at baseline to 8 ms during both LVSP and LBBP. ${IS}_{intra}^{LV}$ also decreased from 79 ms at baseline to 38 ms with LBBP. ${IS}_{intra}^{RV}$ was higher after LBBP (48 ms) compared to LVSP (34 ms) and anodal capture (38 ms).

**Table 1:** Interventricular and intraventricular synchrony markers for each state

${\boldsymbol{I}\boldsymbol{S}}_{\boldsymbol{i}\boldsymbol{n}\boldsymbol{t}\boldsymbol{r}\boldsymbol{a}}^{\boldsymbol{L}\boldsymbol{V}}$	Baseline	RVSP	LVSP	LBBP	LBBP with anodal capture
	79	65	49	38	53
${\boldsymbol{I}\boldsymbol{S}}_{\boldsymbol{i}\boldsymbol{n}\boldsymbol{t}\boldsymbol{r}\boldsymbol{a}}^{\boldsymbol{R}\boldsymbol{V}}$	35	54	34	48	38
${\boldsymbol{I}\boldsymbol{S}}_{\boldsymbol{i}\boldsymbol{n}\boldsymbol{t}\boldsymbol{e}\boldsymbol{r}}^{}$	54	35	8	8	16

Histogram analysis revealed biventricular dyssynchrony with delayed LV activation at baseline, characteristic of LBBB, and a low overlap of 34% (Fig. 2). With RVSP, the overlap increased modestly to 45%, though LV activation remained delayed. Both LVSP and LBBP remarkably improved biventricular synchrony, with greater alignment between RV and LV activation times, reflected by overlaps of 59% and 67%, respectively. Anodal capture also improved biventricular synchrony, with 51% overlap.

**Fig. 3:**
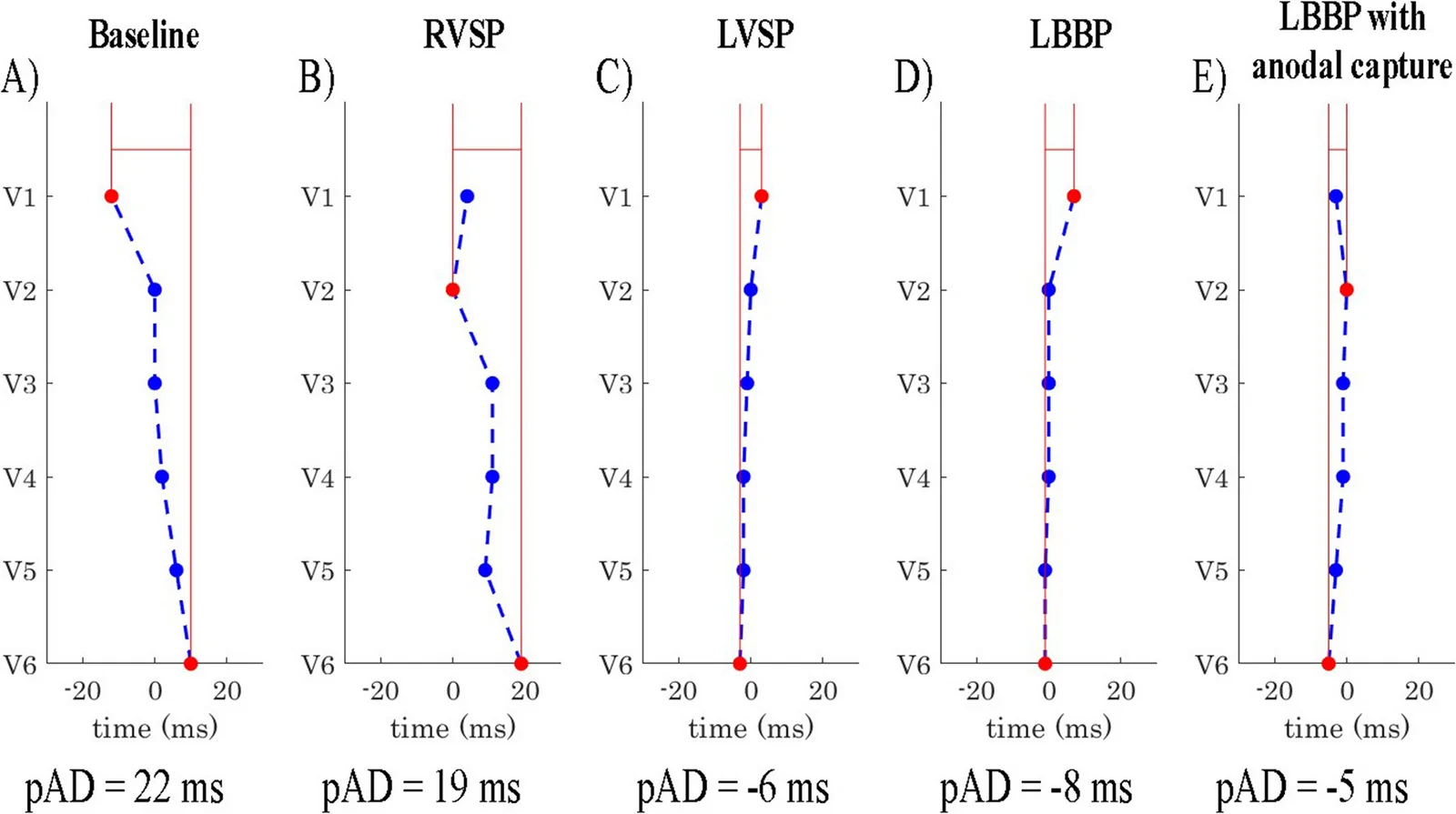
Precordial activation sequences and pAD values for (**A**) baseline, (**B**) RVSP, (**C**) LVSP, (**D**) LBBP and (**E**) LBBP with anodal capture

LF QRS analysis showed that V6 was the latest lead to activate at baseline and after RVSP, with pAD values of 22 ms and 19 ms, respectively. In contrast, LBBP, LVSP, and anodal capture all produced negative pAD values of -6 ms, -8 ms and − 5 ms, indicating reversed activation directionality. V1 was the latest activated lead during LBBP and LVSP, while V2 was the latest one during anodal capture (Fig. 3).

At three-month follow-up, CRT delivered through LBBP achieved a 99% ventricular pacing rate, with improvement in LVEF to 53% and symptoms improved to NYHA Class I.

## Discussion

In this case report, we evaluate ventricular activation patterns during the transition from baseline LBBB to successful LBBP, using both ECGi and LF QRS activation sequence analysis. This real-time assessment provides detailed characterization of inter- and intraventricular synchrony across various pacing modalities.

ECGi-derived epicardial activation maps show effective correction of LBBB with LBBP, consistent with prior studies [8] such as LEVEL-AT trial [9]. Although a single-case study, our work complements existing findings by providing a more comprehensive characterization of each pacing modality using inter- and intraventricular synchrony markers derived from ECGi. Additionally, the estimation of the probability density functions of LV and RV activation times offers a complementary and intuitive visualization of the progressive restoration of interventricular synchrony.

LVSP, LBBP, and anodal capture show comparable AT_t_ and AT_LV_. However, interventricular synchrony, quantified by ${IS}_{inter}^{}$, is more effectively restored with LVSP and LBBP than with anodal capture. This may be attributed to the pseudo-RBBB pattern induced by LVSP and LBBP, which delays median RV activation time and narrows the LV-RV activation difference.

In terms of intraventricular synchrony, LBBP results in more synchronized LV activation than LVSP (lower ${IS}_{intra}^{LV}$). This finding aligns with the significantly shorter V6 RWPT observed during LBBP, likely reflecting direct capture of the specialized conduction system. In contrast, considering ${IS}_{intra}^{RV}$ values, LBBP shows greater electrical dispersion in RV compared to LVSP and anodal capture. This likely reflects delayed RV depolarization, consistent with the longer V6-V1 interpeak interval observed during LBBP compared to LVSP.

Biventricular synchrony, assessed by the overlap between RV and LV activation time distributions, progressively improves from baseline (34%) to RVSP (45%), LVSP (59%), anodal capture (51%), and peaks with LBBP (67%). Delayed RV activation observed with LBBP, relative to LVSP and anodal capture (Fig. 3), likely contributes to this greater temporal overlap, especially given the smaller RV mass compared to the LV.

LF QRS analysis further corroborates these findings. Activation sequences and pAD values progressively improve from baseline LBBB to LBBP. Baseline and RVSP exhibit delayed LV activation, with V1–V2 the earliest activated leads and V6 the latest one, which reflects in positive pAD values (22 and 19 ms, respectively). LVSP and LBBP yield negative small pAD values (-6 ms and − 8 ms, respectively), indicating reversed activation direction consistent with improved electrical synchrony. V1 is the latest activated lead, consistent with the pseudo-RBBB pattern in the ECG. Anodal capture produces near-simultaneous activation of V1 and V6 with negative pAD (-5 ms) and no pseudo-RBBB morphology, indicating a high degree of electrical synchrony comparable to that achieved with LVSP and LBBP. As previously reported [4], pAD does not correlate with V6 RWPT, as it evaluates the dispersion of activation times across leads V1 to V6, encompassing biventricular activation rather than focusing solely on the left ventricle. Nevertheless, some relationships can be observed when compared with the V6–V1 interpeak interval. Absolute pAD values were lower for LVSP compared to LBBP, consistent with the V6-V1 interpeak interval, reflecting the smaller temporal difference between right and left ventricular activation during LVSP compared to LBBP.

Regarding clinical feasibility, a key advantage of the presented metrics is their potential for real-time intraoperative use. ECGi, performed with the ACORYS mapping system, employs a 128-electrode vest to record body-surface signals and estimate cardiac geometry. This system is designed for real-time guidance, as it reconstructs the three-dimensional torso from a rapidly acquired pre-implantation recorded video and estimates cardiac geometry without requiring previous imaging or radiation exposure. As a result, it can generate epicardial maps in real time, which may help guide intraprocedural decision-making regarding optimal lead positioning to achieve adequate electrical resynchronization. Moreover, ECGi-derived and ECG-derived markers require only a few milliseconds of computation, providing immediate feedback comparable to traditional markers such as V6RWPT. However, further integration of signal acquisition into a unified software interface remains essential to ensure these parameters can be seamlessly incorporated into the routine clinical workflow of the electrophysiology laboratory.

As interest grows in differentiating LVSP from LBBP, recent studies [10] suggest similar paced QRSd between the two [11] in patients without structural heart disease, raising questions about their physiological equivalence. Our findings underscore the potential value of ECGi and LF QRS in identifying subtle yet clinically relevant differences in ventricular activation between these two pacing modalities, which should be confirmed by future studies involving larger patient cohorts.

To our knowledge, this is the first report documenting real-time progression of ECG morphologies and ventricular activation patterns from LBBB to successful LBBP using both ECGi and LF QRS methodologies. In line with the 2025 European consensus on conduction system pacing [1], our findings highlight the potential of ECGi as a sophisticated tool for assessing cardiac synchrony. Nevertheless, larger randomized controlled trials are needed to establish the standardized role of ECGi and LF QRS analysis in routine clinical decision-making and to confirm their impact on long-term outcomes.

## Conclusion

This case illustrates the value of ECGi and LF QRS analysis in characterizing and guiding CRT via LBBP. Both techniques provide complementary insights into inter- and intraventricular synchrony and facilitate the comparison between pacing modalities. LVSP, LBBP, and anodal capture all result in more synchronized activation sequences and lower pAD values compared to baseline LBBB, indicating improved electrical synchrony. Among these, LBBP achieves the greatest improvements in interventricular and LV intraventricular synchrony, although it shows higher RV activation dispersion. Although limited to a single observation, these findings provide a hypothesis-generating foundation for the use of ECGi and LF QRS as potential complementary markers in assessing conduction system pacing and guiding CRT personalization. 

## Electronic Supplementary Material

Below is the link to the electronic supplementary material.

Graphical Abstract. Body surface potentials were recorded using a 128-electrode array covering the anterior and posterior torso. Ventricular ECGi maps and derived markers were then computed. A standard 12-lead ECG was simultaneously obtained from 10 conventional electrodes, and the ventricular activation sequence and precordial activation delay (pAD) were calculated. Both baseline and different pacing locations were analyzed.

## Data Availability

The dataset generated and analysed during the current study are not publicly available because they contain sensitive medical information obtained under patient consent, specifically for the purposes of this study, but are available from the corresponding author on reasonable request.

## Declarations

J.R.-M. and M.C.-R. have received consulting fees from Medtronic Iberica S.A. outside the submitted work.
